# Health-related quality of life for patients on hemodialysis in Saudi Arabia: A cross-sectional study

**DOI:** 10.1097/MD.0000000000049784

**Published:** 2026-07-17

**Authors:** Abdullah Hussien Alghamdi, Khalid hamed Alhougail, Sulaiman Abdullah Alraqibah, Reem Hammad Albalawi, Laila Mohammed Mujarribi, Salman Alrasheed, Momen Hashim HajHassan Mohamed, Noor Abdulhakim Alfateel, Faisal Alzkari, Ziyad Alsulami, Musab Alsulami, Rama Abu Hassan, Renad Alsuhaibani, Abdullah Alaryni, Abdulrahman Mohammed Alanazi, Rayan A. Qutob, Abdullah Bukhari, Enad Alsolami, Abdulwahed A. Alotay, Meshal Alzakari

**Affiliations:** a Department of Internal Medicine, College of Medicine, Imam Mohammad Ibn Saud Islamic University (IMSIU), Riyadh, Saudi Arabia; b Department of Medicine, Dr. Sulaiman Al Habib Medical Group, Riyadh, Saudi Arabia; c College of Medicine, Imam Mohammad Ibn Saud Islamic University (IMSIU), Riyadh, Saudi Arabia; d Department of Medicine, College of Medicine, Imam Mohammad Ibn Saud Islamic University (IMSIU), Riyadh, Saudi Arabia; e Department of Internal Medicine, College of Medicine, University of Jeddah, Jeddah, Saudi Arabia.

**Keywords:** chronic kidney disease, hemodialysis, quality of life, Saudi Arabia

## Abstract

The health-related quality of life (HRQoL) for hemodialysis (HD) patients is typically lower than that of kidney transplant patients. Considering that HRQoL is significant for the treatment and well-being of HD patients, this study aimed to evaluate HRQoL among patients undergoing HD in Saudi Arabia. This is a cross-sectional study that was conducted at Dr Sulaiman Al-Habib Hospital in Riyadh, Saudi Arabia, between June and October 2025. Multivariable logistic regression analysis was performed to examine factors associated with physical and mental health outcomes. This study included 93 HD patients. Males had significantly higher PCS (65.72 ± 29.44) and MCS (72.01 ± 23.70) compared to females (PCS: 45.31 ± 29.75; MCS: 56.17 ± 27.18; *P* < .001). Educational level significantly differed with MCS (*P* = .02), with high school graduates and postgraduate degree holders having higher MCS scores. Employment status was also differed in both PCS (*P* < .001) and MCS (*P* = .02). Higher itching in the past 24 hours was associated with lower odds of both PCS (adjusted odds ratio (aOR) = 0.76, 95% confidence intervals [CI]: 0.61–0.96, *P* = .018) and MCS (aOR = 0.64, 95% CI: 0.48–0.85, *P* = .002). for mental health, being Saudi national (aOR = 0.05, 95% CI: 0.00–0.98, *P* = .048), having a high school education (aOR = 17.74, 95% CI: 1.99–158.18, *P* = .010), being a student (aOR = 0.01, 95% CI: 0.00–0.55, *P* = .024), spending 1 to 5 years (aOR = 0.18, 95% CI: 0.03–1.00, *P* = .050), were all associated with higher MCS scores. The current study revealed that males had significantly higher PCS and MCS than females. Hence, considering these findings is essential to developing efficacious targeted interventions to improve HRQoL among patients on HD, particularly those with lower PCS and/or MCS scores. Further studies are required to explain differences in PCS and/or MCS scores among certain patients’ demographic groups.

## 1. Introduction

Chronic kidney disease (CKD) is a prevalent global health problem that affects about 850 million individuals, with an expected increase in CKD cases.^[1–5]^ It is determined by a decrease in renal function or the existence of renal damage for at least 3 months.^[6]^ With progress in CKD, the decreased renal function worsens until permanent renal failure occurs, the last stage of CKD, which is called end stage renal disease, and it usually requires renal replacement therapy (RRT) such as kidney transplantation or dialysis.^[7]^

Peritoneal dialysis and hemodialysis (HD) are the primary types of dialysis treatment.^[8,9]^ HD is more prevalently used for dialysis patients than peritoneal dialysis at the global level (89% vs 11% of patients, respectively). It is also the most widely used RRT, accounting for about 69% of global RRTs.^[8,10–12]^ Although HD enhanced the survival of patients, HD patients suffer from multiple health concerns, including sexual dysfunction, pain, cognitive impairment, emotional distress, strain, fatigue, peripheral neuropathy, and sleep problems.^[13,14]^ Besides, HD is expensive, time-intensive, and in cases of long-term therapy, usually contributes to financial problems, disruption in social life, dependence on caregivers, and loss of freedom. Therefore, it impaired the environmental, socioeconomic, psychological, and physical aspects of patients’ lives.^[15,16]^

Transplant recipients usually expect to return to normal life; however, in reality, patients’ outcomes need continuous monitoring and management using multiple therapeutic approaches. Realistic health expectations are associated with higher satisfaction levels and better quality of life (QoL). Indeed, the health-related quality of life (HRQoL) for dialysis patients is typically lower than that of kidney transplant patients, earlier CKD patients, and the general population.^[17–19]^ Moreover, a prior study reported that the health status of HD patients impacts their HRQoL.^[20]^ Other studies documented a significant correlation between social support from the community, coworkers, family, and friends for patients undergoing HD and QoL for those patients.^[21,22]^ Hence, assessment of HRQoL has become a significant indicator of effectiveness and quality of treatment, patient survival, disease control, prognosis, and consequences.^[23–25]^ Thus, it aids in improving patient outcomes, quality of healthcare, treatment adherence, and making medical decisions based on patients’ social, emotional, and physical needs.^[23]^ Additionally, it is crucial in controlling disease and its outcomes for HD patients.^[25]^ In Saudi Arabia, the annual number of patients on HD increased by 6%.^[26]^ Considering that HRQoL is significant for the treatment and well-being of HD patients, this study aimed to evaluate HRQoL among patients undergoing HD in Saudi Arabia.

## 2. Methods

### 2.1. Study design

This is a cross-sectional study that was conducted at Dr Sulaiman Al-Habib Hospital in Riyadh, Saudi Arabia, between June and October 2025.

### 2.2. Study population

This study was conducted in the HD unit at Dr Sulaiman Al-Habib Hospital in Riyadh, a well-equipped tertiary care center that serves a diverse population of patients with CKD. The study population included adult patients aged 18 years or older undergoing HD.

### 2.3. Study instrument

HRQoL was measured using the Short Form 12-item survey, which includes 12 items assessing physical and mental health domains. Items were coded according to standard Short Form 12-item survey scoring instructions, with higher scores indicating better health status, and the last 2 items were coded reversely. Physical Component Summary (PCS) and Mental Component Summary (MCS) scores were calculated by averaging items relevant to each domain: OCS included general health, physical functioning, role physical, and body pain items, while the MCS domain included vitality, social functioning, role emotional, and mental health items. Both PCS and MCS scores were transformed to a 0 to 100 scale for interpretation, where 0 represents the worst and 100 represents the best possible health.

### 2.4. Ethical approval

This study was approved by the Research Center at Dr Suliman AL Habib Hospital, Riyadh, Saudi Arabia (RC25.11.109). All participants provided their informed consent before participating in this study.

### 2.5. Data analysis

Data were analyzed using the Statistical Package for Social Science version 27. Descriptive statistics were presented as mean and standard deviation for continuous variables and frequency and percentage for categorical variables. Normality of continuous variables such as PCS and MCS was assessed using the Shapiro–Wilk test. Independent *t* test and ANOVA test were applied since the data met the normality assumptions. Multivariable logistic regression analysis was performed to examine factors associated with physical and mental health outcomes, and adjusted odds ratios (aOR) with 95% confidence intervals (CI) were reported. Prior to logistic regression, the scores were dichotomized into high and low levels based on the median of PCS as 62 and MCS as 70. A *P*-value < 0.05 was considered statistically significant for the whole analysis

## 3. Results

This study included 93 HD patients. Among participants, 53 (57.0%) were male and 40 (43.0%) females. The majority were Saudi nationals (84, 90.3%) and residents in the Central region (66, 71.0%). Educational levels varied, with 33 (35.5%) having less than high school, and 16 participants (17.2%) high school graduates. Employment status included (25, 26.9%) employed and 37 participants (39.8%) unemployed, Table 1.

**Table 1:** The sociodemographic characteristics of participants.

Sociodemographic characteristics		N	%
Gender:	Male	53	57.0%
	Female	40	43.0%
Nationality:	Non-KSA	9	9.7%
	KSA	84	90.3%
Region of residence:	Central	66	71.0%
	Western	11	11.8%
	Eastern	3	3.2%
	Northern	2	2.2%
	Southern	11	11.8%
Educational level:	Less than high school	33	35.5%
	High school graduate	16	17.2%
	Diploma	17	18.3%
	Bachelor’s degree	20	21.5%
	Postgraduate degree	7	7.5%
Employment status:	Employed	25	26.9%
	Unemployed	37	39.8%
	Student	2	2.2%
	Retired	29	31.2%

Most participants had been on dialysis for 1 to 5 years (46, 49.5%), followed by <1 year (26, 28.0%). Regarding dialysis access, AVF was used by (40, 43.0%) of participants, while only 1 patient used AVG (1.1%) and (52, 55.9%) reported catheter as dialysis access. Calcium was normal in 47 patients (50.5%), PTH was 14 to 63 pg/mL in 50 patients (53.8%). Using sevelamer, calcium, cinacalcet, and vitamin D was reported by (54, 58.1%), (57, 61.3%), (29, 31.2%), and (55, 59.1%) participants, respectively. The prevalence of severe itching was 10.8% among participants, and the itching scale mean was 2.35 to 2.85 out of 10, with an itching prevalence of 58.1%, Table 2.

**Table 2:** Clinical characteristics and dialysis related factors among study participants.

Clinical characteristics and dialysis related factors		N	%
Yr on dialysis	<1	26	28.0%
	1–5	46	49.5%
	5–10	17	18.3%
	>10	4	4.3%
Access of dialysis	AVF	40	43.0%
	AVG	1	1.1%
	Catheter	52	55.9%
Number of HD sessions missed per mo	1–2	85	91.4%
	3–4	8	8.6%
	5–6	0	0.0%
	>=7	0	0.0%
Hb level	<=8	8	8.6%
	8–10	28	30.1%
	10–12	49	52.7%
	>12	8	8.6%
PO4	Low	7	7.5%
	Normal	47	50.5%
	High	39	41.9%
Ca	Low	44	47.3%
	Normal	47	50.5%
	High	2	2.2%
PTH	<14	11	11.8%
	14–63	50	53.8%
	Above 63	32	34.4%
Kt/v	<1.2	17	18.3%
	1.2–1.4	45	48.4%
	>1.4	31	33.3%
Using of sevelamer	No	39	41.9%
	Yes	54	58.1%
Using of calcium	No	36	38.7%
	Yes	57	61.3%
Using of Cinacalcet	No	64	68.8%
	Yes	29	31.2%
Using any type of Vitamin D	No	38	40.9%
	Yes	55	59.1%
Based on your experience, which group describes your itch severity best?	Mild	71	76.3%
	Moderate	12	12.9%
	Severe	10	10.8%

Mean PCS and MCS scores differed across several participant characteristics. Males had significantly higher PCS (65.72 ± 29.44) and MCS (72.01 ± 23.70) compared to females (PCS: 45.31 ± 29.75; MCS: 56.17 ± 27.18; *P* < .001). Region of residence significantly differed regarding PCS (*P* = .03), the Western region showing the highest PCS (68.56 ± 28.95). Educational level significantly differed with MCS (*P* = .02), with high school graduates and postgraduate degree holders having higher MCS scores. Employment status also differed in both PCS (*P* < .001) and MCS (*P* = .02), Table 3.

**Table 3:** Comparison of physical and mental component summary scores across sociodemographic and clinical characteristics.

Variables		PCS		MCS	
		Mean ± SD	*P*-value	Mean ± SD	*P*-value
Gender	Male	65.72 ± 29.44	.00	72.01 ± 23.70	.00
	Female	45.31 ± 29.75		56.17 ± 27.18	
Nationality	Non-KSA	58.33 ± 36.80	.89	66.30 ± 34.86	.88
	KSA	56.80 ± 30.70		65.08 ± 25.51	
Region of residence	Central	59.60 ± 32.27	.03	67.98 ± 27.58	.16
	Western	68.56 ± 28.95		64.55 ± 23.44	
	Eastern	27.78 ± 4.81		66.67 ± 15.28	
	Northern	12.50 ± 0.00		26.67 ± 18.86	
	Southern	45.45 ± 17.53		55.76 ± 18.74	
Educational level	Less than high school	48.23 ± 32.07	.28	54.75 ± 27.86	.02
	High school graduate	63.28 ± 24.59		77.08 ± 18.21	
	Diploma	63.48 ± 28.28		69.41 ± 23.37	
	Bachelor’s degree	56.46 ± 35.50		64.67 ± 27.47	
	Postgraduate degree	69.05 ± 30.13		78.57 ± 23.87	
Employment status	Employed	73.00 ± 24.12	.00	74.80 ± 19.93	.02
	Unemployed	43.69 ± 26.89		55.50 ± 25.01	
	Student	56.25 ± 55.98		70.00 ± 42.43	
	Retired	60.06 ± 34.19		68.97 ± 29.06	
Yr on dialysis	<1	60.10 ± 33.27	.93	67.69 ± 24.76	.70
	1–5	56.07 ± 27.75		65.00 ± 25.90	
	5–10	54.17 ± 39.56		59.80 ± 31.50	
	>10	58.33 ± 20.69		74.17 ± 21.32	
Access of dialysis	AVF	59.38 ± 28.26	.57	66.83 ± 25.05	.51
	AVG	29.17 ± 0.00		36.67 ± 0.00	
	Catheter	55.61 ± 33.38		64.49 ± 27.42	
Number of HD sessions missed per mo	1–2	55.78 ± 30.60	.24	64.12 ± 25.50	.19
	3–4	69.27 ± 36.05		76.67 ± 33.76	

Multivariable logistic regression analysis showed that several factors were significantly associated with PCS and MCS. Higher itching in the past 24 hours was associated with lower odds of both PCS (aOR = 0.76, 95% CI: 0.61–0.96, *P* = .018) and MCS (aOR = 0.64, 95% CI: 0.48–0.85, *P* = .002). for mental health, being Saudi national (aOR = 0.05, 95% CI: 0.00–0.98, *P* = .048), having a high school education (aOR = 17.74, 95% CI: 1.99–158.18, *P* = .010), being a student (aOR = 0.01, 95% CI: 0.00–0.55, *P* = .024), spending 1 to 5 years (aOR = 0.18, 95% CI: 0.03–1.00, *P* = .050), were all associated with higher MCS scores, Table 4.

**Table 4:** Factors associated with physical and mental health summary scores among dialysis patients (logistic regression).

Variable		PCS		MCS	
		OR (95% CI)	*P*-valuee	OR (95% CI)	*P*-value
Age		1.01 (0.95–1.06)	.851	0.94 (0.89–1.00)	.067
Gender	Male	Reference			
	Female	0.24 (0.03–2.10)	.195	2.82 (0.30–26.20)	.362
Nationality	Non-KSA	Reference			
	KSA	0.96 (0.09–10.10)	.974	0.05 (0.00–0.98)	.048
Region of residence	Central	Reference			
	Western	2.20 (0.38–12.78)	.380	0.64 (0.11–3.61)	.611
	Eastern	0.00 (0.00–0.00)	.999	0.52 (0.02–13.87)	.697
	Northern	0.00 (0.00–0.00)	.999	0.00 (0.00–0.00)	.999
	Southern	0.27 (0.03–2.17)	.220	0.15 (0.01–1.74)	.128
Educational level	Less than high school	Reference			
	High school graduate	2.71 (0.47–15.52)	.261	17.74 (1.99–158.18)	.010
	Diploma	1.32 (0.19–9.07)	.778	4.94 (0.57–42.64)	.146
	Bachelor’s degree	2.43 (0.35–17.03)	.372	2.95 (0.36–24.14)	.314
	Postgraduate degree	0.17 (0.01–4.01)	.274	0.60 (0.02–14.58)	.755
Employment status	Employed	Reference			
	Unemployed	0.44 (0.03–7.30)	.566	0.43 (0.02–10.60)	.607
	Student	0.07 (0.00–2.32)	.137	0.01 (0.00–0.55)	.024
	Retired	0.31 (0.03–3.34)	.335	2.32 (0.21–25.53)	.492
Yr on dialysis	<1	Reference			
	1–5	0.58 (0.15–2.28)	.434	0.18 (0.03–1.00)	.050
	5–10	1.04 (0.14–7.57)	.973	0.07 (0.01–0.80)	.033
	>10	0.33 (0.01–9.45)	.517	0.32 (0.01–12.99)	.548
Access of dialysis	AVF	Reference			
	AVG	0.00 (0.00–0.00)	1.000	0.00 (0.00–0.00)	.999
	Catheter	0.23 (0.05–1.08)	.062	0.03 (0.00–0.27)	.002
Number of HD sessions missed per mo	1–2	Reference			
	3–4	4.36 (0.39–48.83)	0.232	39.55 (2.16–724.58)	.013
Please rate the worst itch you have felt in the previous 24 hours		0.76 (0.61–0.96)	.018	0.64 (0.48–0.85)	.002

## 4. Discussion

The current study showed that males had significantly higher PCS and MCS compared to females. The findings from earlier studies among patients on HD are consistent with this finding. For example, PCS and MCS scores were significantly higher among males than females in Saudi Arabia,^[27]^ Sohag, Southern Albania,^[28]^ Alexandria,^[29]^ and Northern Cyprus.^[30]^ Likewise, male patients had significantly higher PCS than female patients in Greece^[31]^ and South Kerala.^[23]^ Males in Greece^[32]^ and Lahore^[33]^ also reported better overall QoL than females. In the Peruvian city, males showed better HRQoL compared to females.^[34]^ Also, other studies have shown that physical functioning is generally better among males than among females.^[35,36]^ These discrepancies in QoL and HRQoL between males and females may be attributed to several factors, as documented in the literature, including higher adherence to physical activity recommendations,^[35,37]^ better tolerance for kidney disease symptoms,^[30]^ higher financial self-sufficiency,^[34]^ fewer physical conditions related limitations,^[38,39]^ and lower depression, responsibilities, and domestic tasks among males than females, as well as differences in nutrition, lifestyle,^[23,40–45]^ and access to dialysis healthcare services.^[46]^ Hence, considering these factors is essential to developing efficacious gender-specific interventions to improve HRQoL among patients on HD, particularly female patients.

This investigation revealed a significant difference in MCS scores by patients’ educational level; high school graduates and those holding postgraduate degrees had higher MCS scores. In accord with our findings, the emotional well-being score differed by educational status among dialysis patients in the Netherlands.^[47]^ In line with this, previous studies conducted in Palestine,^[48,49]^ Peruvian city,^[34]^ Benha city,^[43]^ Sohag, Southern Albania,^[28]^ and Iran^[50]^ among patients on HD have reported differences in MCS scores by patients’ education levels, and many of these studies also demonstrated differences in PCS^[28,48]^ and other HRQoL measures by patients’ education levels.^[43,50]^ Still, most of these studies found that patients at higher education levels had higher MCS^[34]^ and other HRQoL scores.^[43,48,50]^ Likewise, other earlier studies performed in Palestine ^[51]^, Greece ^[52]^, and Ethiopia^[45]^ among HD patients documented better HRQoL among participants with higher education. On the other hand, MCS was either not significantly affected or not affected by the education status of HD patients in South Kerala,^[23]^ Saudi Arabia,^[27]^ and Malawi.^[53]^ The discrepancies between studies could be due to the effect of demographic factors, including participants’ gender, socioeconomic status, and health status. Regardless of these, research highlighted several reasons for better MCS among higher educational patients or those with a certain degree of education, which include better health literacy,^[47,54–56]^ health-seeking behavior,^[57]^ adherence to dialysis sessions,^[58]^ socioeconomic status,^[47,57]^ accessibility to support,^[59]^ understanding of their condition and related complications,^[58]^ and ability to manage disease-related difficulties.^[59]^ Further studies are required to understand the reasons for the difference in MCS scores between patients on HD with different educational levels. Besides, targeted interventions are necessary to improve mental health among Saudi HD patients based on their level of education.

Our study found that employment status also differed in both PCS and MCS. Previous studies conducted among HD patients have also found differences in HRQoL scores based on employment status; however, most of these studies have demonstrated better HRQoL scores among employed patients. Specifically, mental health and physical functioning scores were higher among employed patients in Kazakhstan^[60]^; psychological and physical QoL domains were higher among employed patients in India^[61]^; PCS and MCS were higher among employed participants in Sohag and Taiwan^[62]^; and HRQoL scores were higher among employed participants in Alexandria.^[29]^ The disparity between our study results and those of previous studies is likely due to other demographic factors, especially income and financial stability. Prior studies among HD patients have shown that economic stability^[48]^ and income^[48,63]^ affect PCS, MCS, and other HRQoL scores by influencing life stress, the ability to afford medications, and access to dialysis services.^[48]^ Thus, studying the effects of income and economic stability may clarify the differences in PCS and MCS by employment status in HD patients in Saudi Arabia.

This study demonstrated that higher itching in the past 24 hours was associated with lower odds of PCS and MCS. Similarly, an earlier multinational investigation observed that PCS and MCS scores were significantly lower among HD patients who were bothered by itchiness compared to those who were not.^[64]^ Another prior study found that itching influences HRQoL in dialysis patients.^[65]^ Moreover, they reported that itching is associated with sleep issues in HD^[64]^ and dialysis patients.^[65]^ Indeed, pruritus is common among CKD patients, and its prevalence increases with progression in the disease.^[66]^ Research documented that the prevalence of pruritus ranges from 40% to 90% among HD patients.^[67–69]^ The intensity of pruritus also affects patients’ work productivity and perceptions of dialysis sessions.^[70]^ Thus, identifying patients with pruritus and managing them effectively are critical to improving HRQoL, survival, and reducing pruritus among these patients.^[68]^

This investigation demonstrated that being a Saudi national, having a high school education, being a student, and spending 1 to 5 years were all associated with higher MCS scores. Having a high school education (education level) and being a student (employment status) could result from different factors, as mentioned earlier; these groups may also experience fewer life stressors. Regarding our finding that Saudi nationals are linked with higher MCS scores, this could be attributed to Saudi religious^[71]^ and cultural beliefs,^[26]^ spiritual coping,^[71]^ and social support,^[72]^ which are reported to be associated with positive outcomes in the country. Indeed, social support is significant for HRQoL of HD patients and for their treatment process and well-being.^[23]^ Consistent with our study, the longer HD duration was associated with lower MCS and/or other HRQoL measures.^[73,74]^ A possible explanation for this is that those HD patients may experience multiple challenges that negatively influence their QoL, including tribulations in treating symptoms, frustration, persistent fatigue, and a repetitive routine.^[45]^ Finally, interventions are required to enhance mental health among Saudi HD patient groups with lower MCS scores.

## 5. Conclusion

The current study revealed that males had significantly higher PCS and MCS than females. A significant difference in MCS scores was observed among patients based on their educational level, and employment status also differed in PCS and MCS scores. Besides, higher itching in the past 24 hours was associated with lower odds of PCS and MCS. On the other hand, being a Saudi national, having a high school education, being a student, and spending 1 to 5 years were all associated with higher MCS scores. Hence, considering these findings is essential to developing efficacious targeted interventions to improve HRQoL among patients on HD, particularly those with lower PCS and/or MCS scores. Further studies are required to explain differences in PCS and/or MCS scores among certain patients’ demographic groups.

## Acknowledgments

This work was supported and funded by the Deanship of Scientific Research at Imam Mohammad Ibn Saud Islamic University (IMSIU) (grant number IMSIU-DDRSP2601).

## Author contributions

**Conceptualization:** Abdullah Hussien Alghamdi.

**Data curation:** Abdullah Hussien Alghamdi.

**Formal analysis:** Abdullah Hussien Alghamdi.

**Funding acquisition:** Abdullah Hussien Alghamdi.

**Investigation:** Abdullah Hussien Alghamdi, Khalid hamed Alhougail, Sulaiman Abdullah Alraqibah, Reem Hammad Albalawi, Laila Mohammed Mujarribi, Salman Alrasheed, Momen Hashim HajHassan Mohamed, Noor Abdulhakim Alfateel, Faisal Alzkari, Ziyad Alsulami, Musab Alsulami, Rama Abu Hassan, Renad Alsuhaibani, Abdullah Alaryni, Abdulrahman Mohammed Alanazi, Rayan A Qutob, Abdullah Bukhari, Enad Alsolami, Abdulwahed A. Alotay, Meshal Alzakari.

**Methodology:** Abdullah Hussien Alghamdi.

**Project administration:** Abdullah Hussien Alghamdi.

**Resources:** Abdullah Hussien Alghamdi, Khalid hamed Alhougail, Sulaiman Abdullah Alraqibah, Reem Hammad Albalawi, Laila Mohammed Mujarribi, Salman Alrasheed, Momen Hashim HajHassan Mohamed, Noor Abdulhakim Alfateel, Faisal Alzkari, Ziyad Alsulami, Musab Alsulami, Rama Abu Hassan, Renad Alsuhaibani, Abdullah Alaryni, Abdulrahman Mohammed Alanazi, Rayan A Qutob, Abdullah Bukhari, Enad Alsolami, Abdulwahed A. Alotay, Meshal Alzakari.

**Software:** Abdullah Hussien Alghamdi.

**Supervision:** Abdullah Hussien Alghamdi.

**Validation:** Abdullah Hussien Alghamdi.

**Visualization:** Abdullah Hussien Alghamdi.

**Writing – original draft:** Abdullah Hussien Alghamdi.

**Writing – review & editing:** Abdullah Hussien Alghamdi, Khalid hamed Alhougail, Sulaiman Abdullah Alraqibah, Reem Hammad Albalawi, Laila Mohammed Mujarribi, Salman Alrasheed, Momen Hashim HajHassan Mohamed, Noor Abdulhakim Alfateel, Faisal Alzkari, Ziyad Alsulami, Musab Alsulami, Rama Abu Hassan, Renad Alsuhaibani, Abdullah Alaryni, Abdulrahman Mohammed Alanazi, Rayan A Qutob, Abdullah Bukhari, Enad Alsolami, Abdulwahed A. Alotay, Meshal Alzakari.
